# Dizziness and Falls in Obese Inpatients Undergoing Metabolic Rehabilitation

**DOI:** 10.1371/journal.pone.0169322

**Published:** 2017-01-11

**Authors:** Stefano Corna, Valentina Aspesi, Nicola Cau, Federica Scarpina, Natalia Gattini Valdés, Luigia Brugliera, Veronica Cimolin, Paolo Capodaglio

**Affiliations:** 1 Rehabilitation Unit and Clinical Lab for Gait Analysis and Posture, Ospedale San Giuseppe, Istituto Auxologico Italiano, IRCCS, Piancavallo (Verbania), Italy; 2 Department of Electronics, Information and Bioengineering, Politecnico di Milano, Milano, Italy; 3 Rita Levi Montalcini” Department of Neuroscience, University of Turin, Turin, Italy; 4 Psychology Research Laboratory, IRCCS Istituto Auxologico Italiano, Ospedale San Giuseppe, Piancavallo (VCO), Italy; 5 Pontificia Universidad Católica de Chile, Physical Medicine and Rehabilitation Resident Universidad de Chile, Santiago, Chile; Children's National Health System, UNITED STATES

## Abstract

**Aim:**

The relationship between dizziness and falls in the obese population is a relatively unexplored issue. The aims of the present study were to define the 1-year prevalence of dizziness in an obese inpatient population undergoing metabolic rehabilitation and to investigate possible correlations with fall events.

**Materials and Methods:**

We recruited 329 obese subjects: 203 female (BMI 43,74 kg/m2 ± 0.5 SE; age 17–83 years, 58.33 ± 0.9 SE) and 126 male (BMI 44,27kg/m2 ± 0.7 DE age 27–79 years, 58.84 ± 1 SE). To assess dizziness we used the validated Italian version (38) of the Dizziness Handicap Inventory (DHI).

**Results:**

Out of the experimental sample, 100 subjects did not complain of dizziness and felt confident about their balance control, while 69.6% reported some degree of dizziness. Their mean DHI score was 22.3, which corresponds to mild dizziness. Twenty-one percent reported more severe dizziness (DHI score > 40). The majority of our sample reported minor dizziness and its perception appears to be independent from BMI: DHI scores were consistent across classes of obesity.

**Discussion:**

The rate of dizziness and falls (30.1%) in an this obese population was higher than that previously reported in a general matched population. However, obese subjects, in our sample, seem to underestimate their risk of fall and DHI score does not appear a reliable predictor of falls. Since complications associated with falls in obese persons generally require longer treatments than in lean individuals, our findings should be taken into account in order to identify other predictors, including cognitive and perceptual, of risk of fall and to implement fall prevention programs.

## Introduction

Dizziness is a common symptom in the general population. Secondary to labyrinth, cardiac, neurological, endocrinological and psychological dysfunctions, dizziness can lead to balance disorders with a significant impact on quality of life and ability to work [1], and can become permanent [2]. Balance disorders increase risk of falls [3], especially in the elderly, with high associated morbidity, mortality, and consequent economic burden [4]. A recent review [5] reports a lifetime prevalence of 17 to 30%. Bisdorff [6] using a more analytic survey for vertigo, dizziness, and unsteadiness, resulting from a range of vestibular and non-vestibular conditions, found a 1- year prevalence of 48.3%, 35.6% and 39.1%, respectively.

Obesity is currently regarded as one of the major health challenges of the developed world and is a growing concern in developing countries. Excessive body weight is an important risk factor for morbidity and mortality from cardiovascular diseases, diabetes, cancer, musculoskeletal and psychiatric disorders with an effect on disability and quality of life [7]. The excessive amount of fat modifies the body’s geometry by adding passive mass to different regions [8], influencing the biomechanics of activities of daily living, causing functional limitations, and possibly predisposing to injury [9]. Quantitative evidence exists that it negatively affects tasks such as sit to stand [10–11], walking [8,10,12] and balance [13]. Adipose tissue accumulation and body mass increase are among the factors contributing to the occurrence of falls and obese persons yield greater risk of fall than normal weight subjects under daily postural stresses and perturbations [14]. Mitchell [15] has recently found that in older age, obesity was associated with a 25% rate of falls in the previous 12 months compared to lean counterparts. The effect of weight on the risk of falling appear to be linear; severe obesity is related to greater risk of falling, although this linearity was not observed with respect to fall-related injury or ADL disability [16]. The relationship between dizziness and falls in the obese population is a relatively unexplored but, given the figures of obesity worldwide, it appears worth exploring it. To our knowledge, only one cross-sectional analysis of a national health survey [17] had reported in obese persons complaining of dizziness a fall event in 35.4% of all the cases, versus 33.7% of a matched lean population. However, the authors acknowledged that their study might have underestimated the prevalence of dizziness due to the nature of their survey.

The aims of the present study were to define the 1-year prevalence of dizziness in an obese inpatient population undergoing metabolic rehabilitation and to investigate possible correlations with fall events.

## Materials and Methods

The Ethics Committee of the Istituto Auxologico Italiano approved this study and subjects provided written informed consent. The latter was provided by the next of kin on behalf of the minors enrolled in the study.

### Subjects

In this study, we recruited 329 obese subjects: 203 female (BMI 43,74 kg/m2 ± 0.5 SE; age 17–83 years, 58.33 ± 0.9 SE) and 126 male (BMI 44,27kg/m2 ± 0.7 DE age 27–79 years, 58.84 ± 1 SE). All subjects were enrolled among those admitted in our Rehabilitation Unit for a metabolic rehabilitation program encompassing weight management, exercise classes, and cognitive-psychological interventions. Our hospital serves as a reference medical rehabilitation center for metabolic conditions in Italy.

All of the subjects underwent first a functional examination by a physiatrist, including joint range of motion, muscle strength and clinical examination, in particular for excluding central and peripheral neurological signs and orthopaedic conditions.

The exclusion criteria were the presence of orthopaedic and neurological disorders (including peripheral neuropathy, visual impairments) and cognitive impairments that may affect the patient´s capacity to understand the questionnaire.

### Experimental Setup

We used the validated Italian version [18] of the Dizziness Handicap Inventory (DHI) [19] (S1 File). A recent review describe it as the most widely used and accepted self-reported measure for dizziness, translated into fourteen languages [20] The DHI was developed to evaluate the self-perceived impairment induced by conditions affecting the vestibular system, but it was also utilized in geriatric, brain injured and multiple sclerosis patients[21–22]. It includes 25 items with a total score ranging between 0 and 100. DHI can be further divided into physical (DHI-p, 28 points), functional (DHI-f 36 points) and emotional (DHI-e 36 points) sub-scores. A higher score indicates a more severe degree of dizziness. The DHI has been reported to have high test-retest reliability, (interclass correlation coefficient [ICC] 0.72–0.97) internal consistency reliability (Cronbach α = 0.72–0.89) and responsiveness (33). The nursing staff distributed and collected questionnaires from 329 subjects who had volunteered to take part in this study. The meaning of the study together with a brief explanation of the scale were reported on the questionnaire’s sheet. DHI was considered suitable for statistical analysis if completed in all items.

Because no data from healthy subject are available in literature, we also collected DHI questionnaire from a group of 40 lean healthy subjects.

According to the WHO, fall is defined as an event which results in a person coming to rest inadvertently on the ground or floor or other lower level (www.who.int/mediacentre/factsheets/fs344/en). A question regarding the presence of fall events in the previous 12 months was added to the questionnaire. To avoid any memory bias, subjects were encouraged to answer “yes” or “no” at this question only when they were adamant and the exact number of falls was not requested.

### Analysis

A T-test was used to discriminate gender-faller/non-faller subjects; in addition an ANOVA and a Bonferroni post-hoc tests were performed between obesity classes.

In order to utilize a continuous scale such as DHI in discriminating subjects with high versus low risk of fall it is necessary to define a cut-off value. The Receiver Operator Curve (ROC) is a plot of sensitivity on the vertical axis and 1-specificity on the horizontal axis for all possible threshold values in the study data set. The information in a ROC curve is summarized as a single value with the most widely used index, the area under the ROC curve (AUC), ranging from 0.5 to 1. A score with no predictive value yields an AUC of 0.5 while a score with complete ability to predict the fall event would have an AUC of 1.

Further analysis to estimate the power of the DHI as predictive tool in respect to the fall event was estimated using likelihood ratio (LR).

## Results

Out of 385 DHI questionnaires collected, 329 were correctly filled and suitable for analysis (Group A) (S2 File). In a subgroup of 196 (Group B), data regarding fall episodes in the previous 12 month were also available (S2 File).

## Analysis A

**Fig 1:**
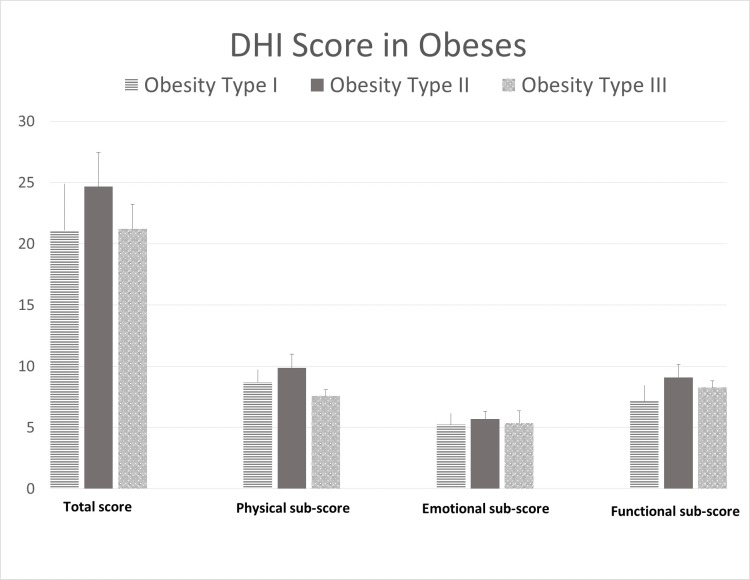
DHI (total and sub-scores) in obese subjects for the three classes.

Group A: 329 obese subjects, 203 female (BMI 43,74 kg/m2 ± 0.5 SE; age 17–83 years, 58.33 ± 0.9 SE) and 126 male (BMI 44,27kg/m2 ± 0.7 DE age 27–79 years, 58.84 ± 1 SE). Non-statistical gender differences were found with respect to age, BMI and DHI (either in total and sub-scores) (p = 0.7 and 0.54, respectively), therefore statistical analysis was performed on a single group of 329 subjects (age 58.5 ± 0.7 SE years, BMI 43,94 kg/m2 ± 0.4 SE). The DHI-total score reported was 22.0 (SE ± 1.4). Sub-scores were DHI-p 8.2 (SE± 0.5), DHI-e 5.4 (SE ± 0.5) and DHI-f 8.4 (SE ± 0.5)–Fig 1.

A lean control group of 40 subjects (26 female), mean age 36.7 years, range 25–60 (SE ± 1.5), BMI 23.4 (SE ± 0.5) also performed the DHI questionnaire. Mean score was 1.8 (SE ± 0.5). The T-test for DHI total score between lean and obese group was significant (p<001). Consequently, we decided to consider symptomatic a DHI score > 2 also for the obese group.

In our obese group, 100 subjects (30.4%) scored between 0 and 2 and 69,6% were considered symptomatic for dizziness. DHI scores were distributed as follows: score 4, 17 subjects; score 6 to 10, 42 subjects; score 12 to 16, 27 subjects; score 18 to 20, 19 subjects; score 22 to 40, 57 subjects and score higher than 42, 69 subjects.

Table 1 shows comparison of age, BMI and DHI (mean value ± SE) for the three obesity classes. Non-statistically significant difference was found between groups.

**Table 1:** Comparison of age, BMI and DHI (mean value ± SE) for the three obesity classes.

	Class I		Class II		Class III		
	Mean value	SE	Mean value	SE	Mean value	SE	ANOVA
Age	56.9	2.8	59.6	1.4	58.3	0.7	n.s.
BMI	32.8	0.2	37.6	0.2	47.7	0.4	< 0.001 (Bonferroni)
DHI—t	21.1	3.6	24.7	3.0	21.2	1.7	n.s.
DHI—p	8.7	1.4	9.9	1.1	7.6	0.5	n.s.
DHI—e	5.2	1.4	5.7	1.0	5.4	0.6	n.s.
DHI—f	7.2	1.4	9.1	1.2	8.3	0.7	n.s.

No correlation was found between age, BMI and DHI scores for either totals and single sub-scores either as a single group or divided in three classes of obesity. Best fit correlation was found for BMI versus DHI-P for class III obese group (r^2^ = 0,0105; r = 0,1025; p = 0,1297; y = 1,54 + 0,1265 x).

## Analysis B

Group B: 196 obese subjects, 131 female and 65 male. Also for this analysis no statistical gender difference was found with respect to age and BMI (p = 0.8 and 0.4, respectively), therefore statistical analysis was performed as single group of 196 subjects of age 58.5 ± 0.9 SE years. BMI was 43.7 kg/m2 ± 0.5 SE. 21 subjects were class I obese, 44 class II and 131 class III. DHI-total score reported was 20.31 (SE ± 1.6). Sub-score were DHI-p 7.7 (SE± 0.5), DHI-e 4.8 (SE ± 0.5) and DHI-f 7.8 (SE ± 0.5).

Fifty-nine subjects were asymptomatic. In 69.9% of the subjects, DHI score was considered symptomatic for dizziness: 38 scored between 4 and 10; 27 between 12 and 20; 34 between 22 and 40 and in 38 more then 40. Out of 196 subjects, 59 (30.1%) reported a fall (3 out of 21 were class I; 14 out of 44 in class II and 42 out of 131 in class III).

Between fallers (F) and non-fallers (NF), t-test failed to find gender, age or BMI differences but found differences with respect to DHI score both in total (F 28.2 ± 3.5 SE; NF 16.84 ± 1.6 SE—p 0,0001) and in sub-scores p (F 10± 1.1 SE; NF 6.7 ± 0.6 SE—p 0,005), e (F 7.4 ± 1.2 SE; NF 3.7 ± 0.5 SE—p 0,002), F(F 10.8 ± 1.4 SE; NF 6.4 ± 0.6 SE—p 0,001). In details: in the asymptomatic group (n 59), the fallers were 24%; in the group with DHI score between 4 and 28 (n 81), the fallers were 33%; in the group with DHI score between 30 an 50 (n 30) the fallers were 30%. In the group with severe (score > 52) dizziness (n 26), the fallers were 50%.

**Fig 2:**
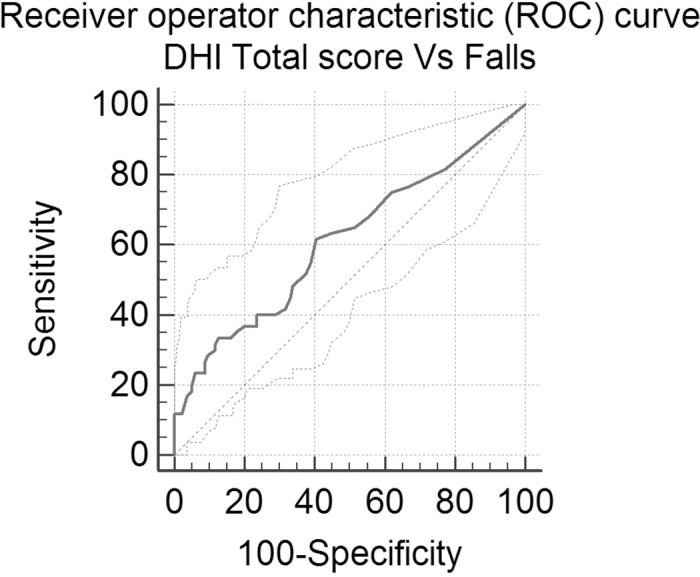
Receiver operating characteristics curve (ROC) of total DHI score prediction model. AUC = 0.612.

In the attempt to determinate a DHI cut-off level with respect to the fall event, we used the Receiver Operating Characteristic (ROC). The score with the highest sensitivity (61.67; 95%Cl 48.2–73.9) and specificity (59.56; 50.8–67.9) was 12. Younden index (sensitivity + specificity—1) J of 0.212; AUC 0.612. Unfortunately the likelihood ratio (LR) analysis demonstrate a low predictive strength of the questionnaire with respect to the fall event. +LR 1.52 (95% Cl1.1–2.0) and the–LR 0.64 (95%Cl 0.5–0.9)–Fig 2.

We also calculated the falls rate (number of subjects/number of falls) at different ages. The rate was low until 50 years of age, substantially increased in the range 50–70 years and decreased again after 70 years (Table 2). The total and sub-total DHI scores showed an increasing but not statistically significant trend with respect to age. In the range 50–70 years, DHI was lower than in the elderly subjects.

**Table 2:** The total and sub-total DHI scores, number of falls an falls-rate (number of subjects/number of falls) in different groups of age.

age interval	N	BMI	DHI-t	DHI-p	DHI-e	DHI-f	falls	falls-rate
< 50	45	44.5	12.6	5.3	2.6	4.6	9	4
51–60	60	44.3	19	7.7	4.2	7.0	20	12
61–70	64	43.4	22.0	7.8	5.4	8.8	22	14.1
>71	28	42	31.8	11.3	8.6	12	8	2.2
ANOVA		n.s.	n.s.	n.s.	n.s.	n.s.		

## Discussion

The risk of fall causes increased disability in ADL [16] and fracture risk [17]. Previous studies had already demonstrated an increased (25%) risk of fall in elderly obese as compared to a lean age-matched population [15]. The novelty of our study was to investigate the prevalence of dizziness in an adult obese population and its relationship with falls. It is known that static balance control is reduced in obese as compared to their lean counterpart [13]. However, the subjective perception of dizziness and its relation with the risk of fall is still a rather neglected area of research. In the general population, the prevalence of dizziness ranges from 17 to 30% [5] and it appears related to the risk of fall [17]. We collected data with a well-known self-reported dizziness scale (DHI) from 329 adult obese (mean age of 58 years, mean BMI of 43.7, female/male ratio of 2:1) subjects free from conditions affecting balance. Because a minimum DHI score level to discriminate between dizzy and non-dizzy subjects is not reported in literature, we tested the questionnaire in a healthy lean population and found a mean score of 2. Based on that, we decided to consider 4 as the threshold score for dizziness. A hundred subjects (30.4%) out of the experimental sample (Group A) did not complain of dizziness and felt confident about their balance control. In this Group, positive responses did not refer to the same items of the questionnaire and dizziness was individually perceived under different conditions. However, the 69.6% prevalence found is noticeably higher than that reported in a general population (17 to 30%) [5]. The overall rating was of mild dizziness (mean score = 22.3), which is close to the 24.5 score previously reported in vestibular loss patients after 2–4 years of unilateral vestibular neurotomy [23]. Dizziness in obesity might be associated with an altered body representation, not only in terms of conscious cognition and perception [24–25], but also in terms unconscious actions (body schema) [26] and sensory perception [27]. In our sample, it was independent from individual body weight. It is possible that the presence of cognitive difficulties in obese subjects [1–6,28] may lead to underestimate the implications of such condition and its health–related consequences.

The analysis on Group B (196 subjects) showed a prevalence of falls of 31%, comparable with that observed in a geriatric population [29]. Although, no data on the general population in their 50ies exist, our data show that around that age the rate of fall dramatically increases. Obesity appears therefore to be *per se* a condition increasing risk of fall. Fall prevention programs must take these results into account.

We used the widely accepted self-reported measure for dizziness DHI as an indicator to define obese subject with greater risk of fall. With the ROC analysis, a score of 12 was found to have a higher sensitivity and specificity level in order to discriminate between fallers and non-fallers. But only in the group with a very high score (> 52) the number of fallers was relevant and the likelihood ratio showed only a marginal utility of DHI in discriminating fallers in the obese population.

### Limitation of the Present Study

Our population is in-patient. However, the mission of our Institute is the clinical and rehabilitation management of obesity and metabolic conditions, therefore, patients with no other conditions than obesity can be hospitalized. Generalization of our results, however, may be hindered by possible modifications in balance self-perception due to the restriction of movement related to a non-habitual environment.

## Conclusions

The rates of dizziness and falls in an inpatient obese population appear higher than in a general matched population. Dizziness and falls do not seem related to the severity of obesity. Obese subjects seem to underestimate their risk of fall and DHI score does not appear to reliably predict falls. Since complications associated with falls often require longer treatment in the obese than in lean individuals, our findings should be taken into account in order to identify other predictors, including cognitive and perceptual, of risk of fall and to implement fall prevention programs.

## Supporting Information

S1 File Dizziness Handicap Inventory-English.pdf. (PDF)

S2 File Data.xls. (XLS)

## References

[1] Yardley L, Owen N, Nazareth I, Luxon L. Prevalence and presentation of dizziness in a general practice community sample of working age people. Br J Gen Pract. 1998; 48: 1131–1135. 9667086 PMC1410052

[2] Skoien AK, Wilhemsen K, Gjesdal S. Occupational disability caused by dizziness and vertigo: a register-based prospective study. BrJ Gen Pract. 2008;58: 619–623. 18801279 10.3399/bjgp08X330744 PMC2529199

[3] Agrawal Y, Carey JP, Della Santina CC, Schubert MC, Minor LB. Disorders of balance and vestibular function in US adults: data from the National Health and Nutrition Examination Survey. Arch Intern Med. 2004;169: 938–944. 10.1001/archinternmed.2009.66 19468085

[4] Siracuse JJ, Odell DD, Gondek SP, Odom SR, Kasper EM, Hauser CJ, et al Health care and socioeconomic impact of falls in the elderly. Am J Surg. 2012;203: 335–338. 10.1016/j.amjsurg.2011.09.018 22257741

[5] Murdin L, Schilder AG. Epidemiology of Balance Symptoms and Disorders in the Community: A Systematic Review. Otol Neurotol. 2015;36: 387–392. 10.1097/MAO.0000000000000691 25548891

[6] Bisdorff A, Bosser G, Gueguen R, Perrin P. The epidemiology of vertigo, dizziness, and unsteadiness and its links to co-morbidities. Front Neurol. 2013;22: 1–7. 10.3389/fneur.2013.00029 PMC3605504 23526567

[7] Bray GA. Medical consequences of obesity. J Clin Endocrinol Metab. 2004;89: 2583–2589. 10.1210/jc.2004-0535 15181027

[8] De Souza SA, Faintuch J, Valezi AC, Sant' Anna AF, Gama-Rodrigues JJ, De Batista Fonseca IC, et al Gait cinematic analysis in morbidly obese patients. Obes Surg. 2005;15: 1238–1242. 10.1381/096089205774512627 16259878

[9] Wearing SC, Hennig EM, Byrne NM, Steele JR, Hills AP. The biomechanics of restricted movement in adult obesity. Obes Rev. 2006;7: 13–24. 10.1111/j.1467-789X.2006.00215.x 16436099

[10] Sibella F, Galli M, Romei M, Montesano A, Crivellini M. Biomechanical analysis of sit-to-stand movement in normal and obese subjects. Clin Biomech. 2003;18: 745–750. 10.1016/s0268-0033(03)00144-x 12957561

[11] Galli M, Crivellini M, Sibella F, Montesano A, Bertocco P, Parisio C. Sit-to-stand movement analysis in obese subjects. Int J Obes. 2000;24: 1488–1492. 10.1038/sj.ijo.0801409 11126346

[12] Vismara L, Romei M, Galli M, Montesano A, Baccalaro G, Crivellini M, Grugni G. Clinical implications of gait analysis in the rehabilitation of adult patients with “Prader-Willi” Syndrome: a crossectional comparative study (“Prader-Willi” Syndrome vs matched obese patients and healthy subjects). J Neuroengineering Rehabil. 2007; 4:14. 10.1186/1743-0003-4-14 PMC1872029 17493259

[13] Menegoni F, Galli M, Tacchini E, Vismara L, Cavigioli M, Capodaglio P. Gender-specific effect of obesity on balance. Obesity. 2009;17: 1951–1956. 10.1038/oby.2009.82 19325540

[14] Finkelstein EA, Chen H, Prabhu M, Trogdon JG, Corso PS. The relationship between obesity and injuries among U.S. adults. Am J Health Promot. 2007;21: 460–468. 17515011 10.4278/0890-1171-21.5.460

[15] Mitchell RJ, Lord SR, Harvey LA, Close JC. Obesity and falls in older people: Mediating effects of disease, sedentary behavior, mood, pain and medication use. Arch Gerontol Geriatr. 2015; 60: 52–58. 10.1016/j.archger.2014.09.006 25307955

[16] Himes CL, Reynolds SL. Effect of Obesity on Falls, Injury, and Disability. J Am Geriatr Soc. 2012;60: 124–129. 10.1111/j.1532-5415.2011.03767.x 22150343

[17] Premaor MO, Comim FV, Compston JE. Obesity and fractures. Arq Bras Endocrinol Metabol. 2014;58: 470–477. 25166037 10.1590/0004-2730000003274

[18] Nola G, Mostardini C, Salvi C, Ercolani AP, Ralli G. Validity of Italian adaptation of the Dizziness Handicap Inventory (DHI) and evaluation of the quality of life in patients with acute dizziness. Acta Otorhinolaryngol Ital. 2010;30:190 21253284 PMC3008147

[19] Jacobson GP, Newman CW. The development of the Dizziness Handicap Inventory, Arch Otolaryngol Head Neck Surg. 1990;116: 424–427. 2317323 10.1001/archotol.1990.01870040046011

[20] Mutlu B, Serbetcioglu B, Discussion of the dizziness handicap inventory. J Vestib Res. 2013; 23: 271–277. 10.3233/VES-130488 24447966

[21] Whitney S L, Hudak MT, Marchetti GF. The activities-specific balance confidence scale and the dizziness handicap inventory: a comparison. J Vestib Res. 1999;9: 253–259. 10472037

[22] Cattaneo D, Jonsdottir J, Repetti S. Reliability of four scales on balance disorders in persons with multiple sclerosis. Disabil Rehabil. 2007;29: 1920–1925. 10.1080/09638280701191859 17852286

[23] Young L, Bernard-Demanze L, Dumitrescu M, Magnan J, Borel L, Lacour M. Postural performance of vestibular loss patients under increased postural threat. J Vestib Res 2012;22: 129–138. 10.3233/VES-2012-0449 23000612

[24] Docteur A, Urdapilleta I, Defrance C, Raison J. Body perception and satisfaction in obese, severely obese, and normal weight female patients. Obesity. 2010;18: 1464–1465. 10.1038/oby.2009.418 19910933

[25] Cafri G, Thompson JK. Measuring Male Body Image: A Review of the Current Methodology. Psychol. Men Masculin. 2004;5: 18–29.

[26] Scarpina F, Castelnuovo G, Molinari E. Tactile mental body parts representation in obesity. Psychiatry Res. 2014;220: 960–969. 10.1016/j.psychres.2014.08.020 25312390

[27] Peltonen M, Lindroos AK, Torgerson JS. Musculoskeletal pain in the obese: a comparison with a general population and long-term changes after conventional and surgical obesity treatment. Pain. 2003;104: 549–57. 12927627 10.1016/S0304-3959(03)00091-5

[28] Yardley L, Masson E, Verschuur C, Haacke N, Luxon L. Symptoms, anxiety and handicap in dizzy patients: development of the vertigo symptom scale. J. Psychosom. Res. 1992; 36: 731–741. 1432863 10.1016/0022-3999(92)90131-k

[29] Hausdorff JM, Rios DA, Edelberg HK. Gait variability and fall risk in community-living older adults: a 1-year prospective study. Arch Phys Med Rehabil. 2001;82: 1050–1056. 10.1053/apmr.2001.24893 11494184

