# Supplementary material for: Dizziness and Falls in Obese Inpatients Undergoing Metabolic Rehabilitation
Source: PLoS One. 2017 Jan 11;12(1):e0169322. doi: 10.1371/journal.pone.0169322 (PMC5226725; doi:10.1371/journal.pone.0169322)
Supplement: S1 File — (PDF) [file pone.0169322.s001.pdf]

The Dizziness Handicap Inventory (DHI)

P1. Does looking up increase your problem?	o Yes<br>o Sometimes<br>o No
E2. Because of your problem, do you feel frustrated?	o Yes<br>o Sometimes<br>o No
F3. Because of your problem, do you restrict your travel for business or recreation?	o Yes<br>o Sometimes<br>o No
P4. Does walking down the aisle of a supermarket increase your problems?	o Yes<br>o Sometimes<br>o No
F5. Because of your problem, do you have difficulty getting into or out of bed?	o Yes<br>o Sometimes<br>o No
F6. Does your problem significantly restrict your participation in social activities, such as going out to dinner, going to the movies, dancing, or going to parties?	o Yes<br>o Sometimes<br>o No
F7. Because of your problem, do you have difficulty reading?	o Yes<br>o Sometimes<br>o No
P8. Does performing more ambitious activities such as sports, dancing, household chores (sweeping or putting dishes away) increase your problems?	o Yes<br>o Sometimes<br>o No
E9. Because of your problem, are you afraid to leave your home without having someone accompany you?	o Yes<br>o Sometimes<br>o No
E10. Because of your problem have you been embarrassed in front of others?	o Yes<br>o Sometimes<br>o No
P11. Do quick movements of your head increase your problem?	o Yes<br>o Sometimes<br>o No
F12. Because of your problem, do you avoid heights?	o Yes<br>o Sometimes<br>o No
P13. Does turning over in bed increase your problem?	o Yes<br>o Sometimes<br>o No
F14. Because of your problem, is it difficult for you to do strenuous homework or yard work?	o Yes<br>o Sometimes<br>o No
E15. Because of your problem, are you afraid people may think you are intoxicated?	o Yes<br>o Sometimes<br>o No
F16. Because of your problem, is it difficult for you to go for a walk by yourself?	o Yes<br>o Sometimes<br>o No
P17. Does walking down a sidewalk increase your problem?	o Yes<br>o Sometimes<br>o No
E18.Because of your problem, is it difficult for you to concentrate	o Yes<br>o Sometimes<br>o No
F19. Because of your problem, is it difficult for you to walk around your house in the dark?	o Yes<br>o Sometimes<br>o No

E20. Because of your problem, are you afraid to stay home alone?	o Yes<br>o Sometimes<br>o No
E21. Because of your problem, do you feel handicapped?	o Yes<br>o Sometimes<br>o No
E22. Has the problem placed stress on your relationships with members of your family or friends?	o Yes<br>o Sometimes<br>o No
E23. Because of your problem, are you depressed?	o Yes<br>o Sometimes<br>o No
F24. Does your problem interfere with your job or household responsibilities?	o Yes<br>o Sometimes<br>o No
P25. Does bending over increase your problem?	o Yes<br>o Sometimes<br>o No

Used with permission from GP Jacobson.
Jacobson GP, Newman CW: The development of the Dizziness Handicap Inventory. *Arch Otolaryngol Head Neck Surg* 1990;116: 424-427

DHI Scoring Instructions

The patient is asked to answer each question as it pertains to dizziness or unsteadiness problems, specifically considering their condition during the last month. Questions are designed to incorporate functional (F), physical (P), and emotional (E) impacts on disability.

To each item, the following scores can be assigned:
No=0 Sometimes=2 Yes=4

Scores:
Scores greater than 10 points should be referred to balance specialists for further evaluation.

16-34 Points (mild handicap)
36-52 Points (moderate handicap)
54+ Points (severe handicap)
